# Socio‐economic inequalities in the effectiveness of workplace health promotion programmes on body mass index: An individual participant data meta‐analysis

**DOI:** 10.1111/obr.13101

**Published:** 2020-07-21

**Authors:** Suzan J.W. Robroek, Karen M. Oude Hengel, Allard J. van der Beek, Cécile R.L. Boot, Frank J. van Lenthe, Alex Burdorf, Pieter Coenen

**Affiliations:** ^1^ Department of Public Health Erasmus University Medical Center Rotterdam The Netherlands; ^2^ Department of Work Health Technology Netherlands Organisation for Applied Scientific Research TNO Leiden The Netherlands; ^3^ Department of Public and Occupational Health, Amsterdam UMC, Vrije Universiteit Amsterdam Amsterdam Public Health Research Institute Amsterdam The Netherlands

**Keywords:** inequity, obesity, socio‐economic inequalities, workplace

## Abstract

This individual participant data meta‐analysis assessed the effectiveness of workplace health promotion programmes on body mass index (BMI) across socio‐economic groups and whether study and intervention characteristics explained inequalities in effectiveness. Studies were eligible if they assessed the effect of a workplace health promotion programme on BMI in the Netherlands, included workers of at least two different socio‐economic positions (SEPs) and had a study design with premeasurement and postmeasurement and control condition. Data of 13 studies presenting 16 interventions (5183 participants) were harmonized. In a two‐stage meta‐analysis, the interaction between intervention and SEP on BMI was tested with linear mixed models for each study. Subsequently, the interaction terms were pooled. The influence of study and intervention characteristics on the effectiveness of workplace health promotion programmes was evaluated using meta‐regression analyses. Compared with control conditions, workplace health promotion programmes overall showed a statistically non‐significant 0.12 kg/m^2^ (95% CI: −0.01, 0.25) decrease in BMI, which did not differ across SEP. Interventions evaluated within randomized controlled trials, agentic interventions, those that focused on high‐risk groups, included a counselling component, consisted of more than five sessions, or were offered at the individual level did statistically significantly reduce BMI. No evidence was found for intervention‐generated SEP inequalities.

**Abbreviations**
BMI: body mass index
IPD: individual participant data
RCT: randomized controlled trial
SEP: socio‐economic position

## 1 INTRODUCTION

Large socio‐economic inequalities in obesity exist among adults in Western countries.^1^ This can partly be explained by a more unhealthy diet and lower physical activity levels among low socio‐economic groups than among higher socio‐economic groups.^2^, ^3^ In the past decades, numerous health promotion programmes have been developed to improve these health behaviours and to prevent obesity. However, there are concerns that health promotion programmes might increase, rather than reduce, inequalities due to a higher reach and/or effectiveness among individuals with a high socio‐economic position (SEP) compared with those with a low SEP.^4^

Only limited information is available about the differential effectiveness of public health interventions across socio‐economic groups.^5^, ^6^ In several reviews, the majority of the included studies did not find differential effects of public health interventions targeting health behaviour across socio‐economic groups.^5^, ^6^, ^7^, ^8^, ^9^, ^10^ According to a framework for the likely impact of obesity prevention strategies on socio‐economic inequalities in body weight, interventions can be categorized based on the degree to which an intervention involves the capacity of individuals to make independent, purposive choices (i.e., individual agency).^11^ This framework distinguishes agentic interventions (high individual agency), agento‐structural interventions (some individual agency) and structural interventions (no individual agency). It is hypothesized that higher socio‐economic groups will benefit more from interventions with a higher level of individual agency.^11^ Indeed, some studies showed that agentic interventions, such as health education or counselling programmes or mass media campaigns, could widen socio‐economic inequalities in health behaviour or health.^5^, ^6^, ^9^, ^11^ This is in contrast with structural interventions, such as removing unhealthy food options or fiscal interventions, which facilitate healthier choices and may contribute to reducing inequalities.^6^, ^9^, ^11^ Agento‐structural interventions do consider the environment, but individual agency is still important, for example, in interventions providing healthier food options in canteens.^11^

Health promotion activities can be implemented in different settings. The workplace has been identified as a promising setting for health promotion due to the substantial time adults spent at work and the ability to reach large groups of participants in a natural social network. A recent review has shown positive, but small, effects of workplace health promotion programmes on body mass index (BMI).^12^ Because workplace health promotion programmes can vary in the degree of agentic involvement, ranging from entirely agentic (such as health education or counselling programmes) to structural interventions (such as removal of vending machines containing unhealthy food and drink options), understanding the equity impact is highly relevant. However, information on intervention‐generated inequalities for workplace health promotion programmes is scarce. Overall, the majority of studies included in previous reviews on workplace health promotion programmes did not find differential effectiveness across socio‐economic groups.^8^, ^13^, ^14^, ^15^ Yet most reviews compared workplace health promotion programmes provided to the general working population with those targeted to blue collar workers or workers with a lower SEP only.^13^, ^14^ Equity‐specific subgroup analysis within specific interventions can contribute to understanding the differential effectiveness of interventions, which is possible in an individual participant data (IPD) meta‐analysis. An IPD meta‐analysis furthermore provides the opportunity to investigate which type of studies and interventions could contribute to reducing socio‐economic inequalities in BMI. As described above, it is hypothesized that agentic interventions will increase socio‐economic inequalities in BMI.

The current IPD meta‐analysis enables analyses that go beyond those that have been performed in the original studies and conventional meta‐analyses. The current study contributes to the existing literature in two ways. First, more in‐depth insight into the differential effects within and across workplace health promotion programmes can be assessed with equity‐specific subgroup analyses. Second, the influence of study design and intervention characteristics on these differential effects can be studied, which contributes to the understanding of the influence of specific study and intervention components on the effectiveness of workplace health promotion programmes. This IPD meta‐analysis will be performed in the Dutch context, which enables to study the effectiveness of the interventions in heterogeneous populations in a rather homogeneous occupational health and social security context. The study aims to investigate the differential effects of workplace health promotion programmes on BMI between socio‐economic groups and the extent to which study and intervention characteristics explain possible differences in effectiveness across groups.

## 2 METHODS

### 2.1 Search strategy and selection of studies

This IPD meta‐analysis was performed according to the earlier published protocol,^16^ which was also registered with PROSPERO (CRD42018099878). The PRISMA‐IPD guidelines were used for reporting our findings. As described in detail in the protocol paper, a systematic search was performed to identify relevant studies aimed at promoting healthy behaviour or preventing obesity among workers. The current paper evaluates differential effectiveness of workplace health promotion programmes on BMI. Another paper, using the same generic data set, evaluates differential effectiveness on health behaviours. The search was restricted to Dutch published and unpublished studies, ensuring that all participants are part of the same occupational health and social security system. Search terms included health behaviour, obesity, intervention, evaluation and worker/worksite. The full search strategy can be found in Supplementary file A. The search for published studies was performed in February 2018 in the following electronic databases: Embase, Medline Ovid, Web of Science, Cochrane Central and Google Scholar. In addition, reference lists of relevant systematic reviews were screened. The search for unpublished studies was conducted through screening of trial registers, databases of major Dutch funding agencies, a Dutch database for lifestyle interventions and consultation of experts.

Inclusion criteria for this specific paper were (i) an intervention study aimed at promoting healthy behaviour or preventing obesity, (ii) targeted at workers, (iii) performed in the Netherlands, (iv) from a study design with a reference group and at least one premeasurement and postmeasurement of BMI and (v) having an indicator for SEP (i.e., educational level, job title or income). Interventions on workers from a clinical sample were excluded, as well as interventions with participants from a single socio‐economic group. No restrictions were made concerning the reference group. Two authors (S.R. and P.C.) screened titles, abstracts and if required full texts of all references for eligibility. A third author (K.O.H.) was consulted in case of disagreement.

For each eligible study, the corresponding author was contacted with a request to sign a data sharing agreement and to share their anonymized IPD. The Medical Ethical Committee of Erasmus MC Rotterdam declared that the Medical Research Involving Human Subjects Act does not apply to the current IPD meta‐analysis (MEC‐2018‐1143).

### 2.2 Data extraction

For each included study, data on study design and intervention characteristics were extracted by one author (S.R., K.O.H. or P.C.) and verified by another author (S.R., K.O.H. or P.C.). Study characteristics included the study design, categorized into randomized controlled trial (RCT), cluster RCT, and CT. Intervention characteristics included the study sample, intervention components, type of intervention delivery, number of sessions and the level of the intervention. For the study sample, a distinction was made between interventions provided to all employees (universal prevention) or interventions targeted to individuals with a high risk, such as individuals with a high BMI or with unhealthy behaviours (selected/indicated prevention). In accordance with the framework for the likely impact of obesity prevention strategies on socio‐economic inequalities in population weight, agentic, agento‐structural and structural interventions were distinguished. Concerning the intervention components, a distinction was made between interventions with or without a counselling component and between interventions with or without an environmental intervention component. The type of delivery was categorized as including a face‐to‐face component versus other (e.g., e/m‐health or environmental changes). The number of sessions was dichotomized into studies with more than five sessions and those with five or less sessions. In addition, interventions focused on the individual level were distinguished from those at group level.

### 2.3 Methodological quality

Methodological quality was also assessed by one author (S.R., K.O.H. or P.C.) and verified by another author (S.R., K.O.H. or P.C.). As previously used in another systematic review,^17^ a combination of a checklist based on the guidelines of the Cochrane Collaboration's tool for assessing risk of bias and the checklist applied by Verweij et al. was used.^18^ This consisted of nine criteria regarding randomization, blinding of participants, similarity of groups, compliance, loss to follow‐up, intention‐to‐treat, adjustment for confounders, data collection methods and follow‐up duration.^17^ On each item, a study could score positive if the quality criterion was met (1 point), negative if the criterion was not met (0 points) or unclear if the publication and/or an additional information request by authors provided insufficient information to make a judgment (0 points). Summary scores were categorised as poor (0–2 points), fair (3–4 points), good (5–7 points) or excellent (8–9 points). Both the data extraction form and methodological quality scale were sent to the corresponding author of the original study for verification.

### 2.4 Harmonization

Data from all studies were harmonized. If a study contained more than one intervention arms, these arms were all considered as separate interventions. In case of more than one control arms, these arms were combined into one control group. All information from the included studies, both the harmonized IPD and the data extracted from the original articles, were merged into a single dataset.

### 2.5 Body mass index

A continuous measure of BMI (kg/m^2^), obtained from self‐reports or objective measures, was used from pre‐intervention and post‐intervention measurements. The measurements could be assessed directly after the intervention (immediate effects) or after a longer follow‐up period (sustained effects). The timing of these measurements differed between studies.

### 2.6 Socio‐economic position

Most interventions included education as indicator of SEP, which was divided into low (pre‐primary, primary and lower secondary education), intermediate (upper secondary education) and high (post‐secondary education), based on the 1997 International Standard Classification of Education (ISCED‐97). In one study, where information on educational level was lacking, occupational class was used to define SEP.^19^ Here, among workers from a construction company, the construction workers were categorised as low SEP and the office workers as intermediate SEP.

### 2.7 Covariates

As in the original studies, age was used as a continuous variable and gender was dichotomized into male and female.

### 2.8 Statistical analysis

A two‐stage meta‐analysis approach was performed. In the first stage, IPD data of each study were analysed separately using multilevel linear mixed models. In the second stage, the results per study were pooled in a meta‐analysis. In the first stage, a random intercept for participant was used, and, for studies with a clustered design, a random intercept for cluster was added to take into account the clustering of participants. Overall effects and interaction effects with SEP (intervention * SEP) were analysed, and all models were stratified by SEP. In case a SEP group in an included study consisted of less than 10 participants, no subgroup analysis or interaction analysis was performed for that specific SEP group in that particular study. For the two studies without any workers with a high SEP,^19^, ^20^ the effects among workers with a low SEP were compared with workers with an intermediate SEP. As no statistically significant intervention * time interaction effects were found, both immediate and sustained effects were added jointly in the mixed model. All models were adjusted for baseline BMI, age and gender.

Meta‐regression analyses were performed to assess the univariable influence of the study and intervention characteristics on the effectiveness and differential effectiveness of workplace health promotion programmes, as well as on the effectiveness stratified by socio‐economic group.

Statistical analyses were conducted using Stata (version 14, *mixed* command for the linear mixed model, *admetan* command for the meta‐analyses and *metareg* command for the meta‐regression). Review Manager (version 5.3.5) was used to draw forest plots, depicting individual study effect sizes. The level of statistical significance was set at *p* < 0.05.

## 3 RESULTS

**FIGURE 1:**
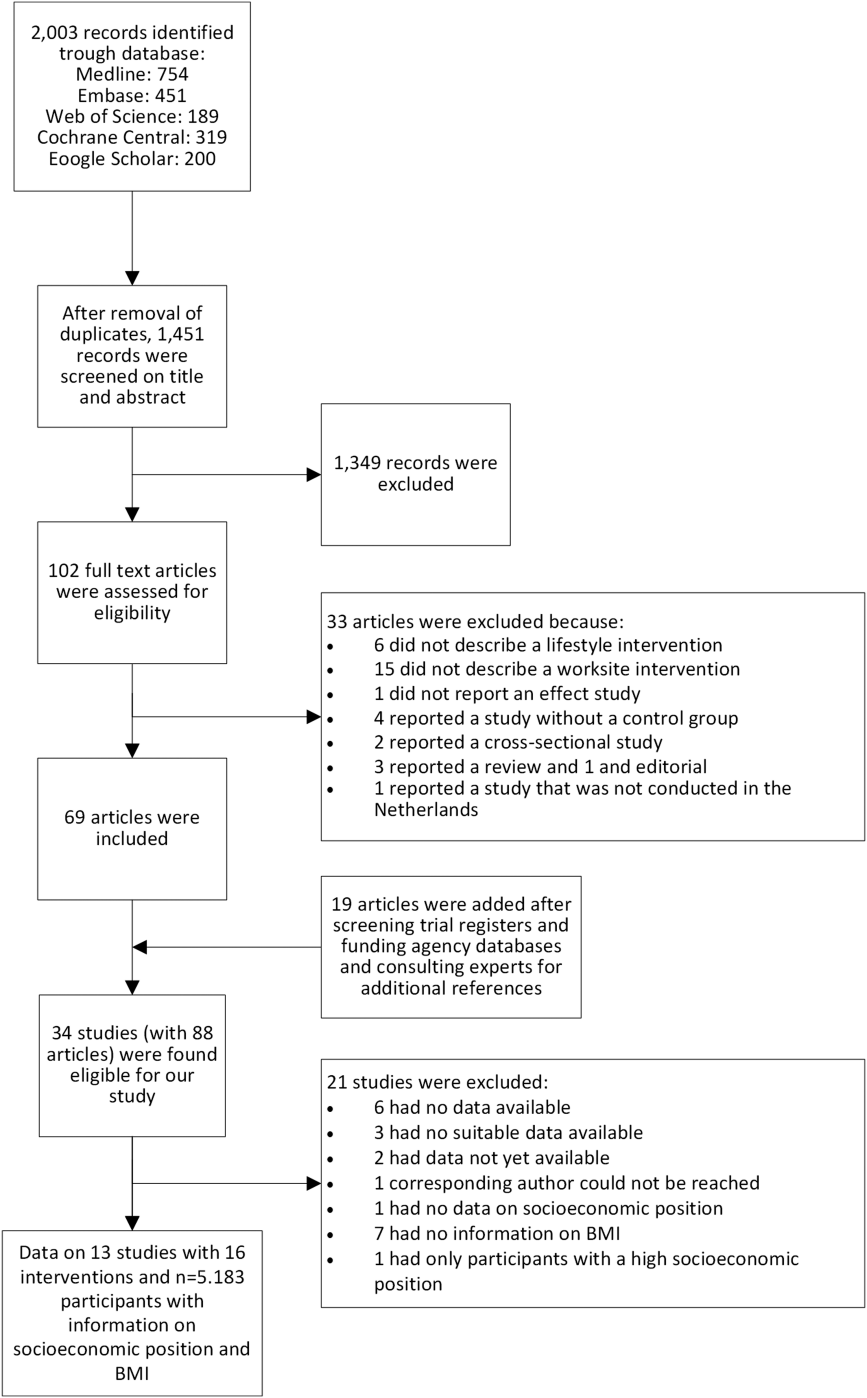
Flow chart of study inclusion process

In total, 34 studies with 88 articles out of the 1415 screened articles were considered eligible for the current study of which 21 studies were excluded due to the unavailability of the data (*n* = 11), the absence of information on BMI as outcome (*n* = 7), lacking information on SEP (*n* = 1), no response from the corresponding author or other involved researchers (*n* = 1) or not considering workers from multiple socio‐economic groups (*n* = 1). Supplementary file B presents the references of these excluded studies. Data of 5183 participants from 13 studies were analysed in the current IPD meta‐analyses (Figure 1). Two studies evaluated more than one intervention arm,^21^, ^22^ which resulted in a total of 16 interventions.

The methodological quality of all studies, except one,^23^ was judged ‘good’ or ‘excellent’ (Table 1). Six studies concerned an RCT,^19^, ^20^, ^22^, ^24^, ^25^, ^26^ four a cluster RCT with, for example, the occupational physician or department as cluster,^21^, ^27^, ^28^, ^29^ and three interventions were evaluated in a CTs.^23^, ^30^, ^31^ Most interventions (*n* = 11) were agentic interventions, five had a combination of agentic and agento‐structural intervention elements, and there were no structural interventions. Twelve interventions included a counselling component,^19^, ^20^, ^21^, ^22^, ^23^, ^24^, ^26^, ^27^, ^28^, ^29^ whereas six interventions included (also) a change in the work environment (such as free or healthy food options at work, or signs to promote stair use)^21^, ^30^, ^31^ (Table 1). Ten interventions consisted of universal prevention strategies,^20^, ^21^, ^23^, ^24^, ^26^, ^28^, ^30^, ^31^ whereas six interventions were offered to high‐risk workers, that is, workers with an unhealthy behaviour, high BMI or high cardiovascular risk.^19^, ^22^, ^25^, ^27^, ^29^ There was overlap between study and intervention characteristics: interventions focused on a high‐risk group were all individual‐level interventions, while this was the case for only three out of the 10 universal interventions. Moreover, interventions focused on a high‐risk group more often had more than five sessions compared with universal prevention strategies.

**TABLE 1:** Main characteristics of studies included in this individual participant data (IPD) meta‐analysis

First author, year	Design^a^	Study population characteristics				Intervention characteristics							Control condition	Overall effect	Methodological quality
		*n* ^b^	Socio‐economic position (%)			Intervention type^c^	Indicated prevention	Counselling component	Environmental component	Individual level	>5 sessions	Face‐to‐face contact	
			Low	Intermediate	High									*Β* (SE)	Sum score
van Berkel, 2014^24^	RCT	222	2	17	81	A‐S	‐^d^	X^d^	X	‐	X	X	Generic information	0.00 (0.12)	8 (excellent)
Coffeng, 2014^21^	cRCT	310	2	42	56	Arm 1: ‐ Arm 2: A‐S Arm 3: A‐S	Arm 1: ‐ Arm 2: ‐ Arm 3: ‐	Arm 1: X Arm 2: ‐ Arm 3: X	Arm 1: ‐ Arm 2: X Arm 3: X	Arm 1: ‐ Arm 2: ‐ Arm 3: ‐	Arm 1: ‐ Arm 2: ‐ Arm 3: ‐	Arm 1: X Arm 2: ‐ Arm 3: X	No intervention	Arm 1: −0.05 (0.14) Arm 2: 0.15 (0.14) Arm 3: −0.01 (0.15)	7 (good)
Engbers, 2007^30^	CT	446	3	30	67	A	‐	‐	X	‐	‐	‐	No intervention	0.12 (0.11)	6 (good)
Groeneveld, 2010^19^	RCT	713	76	24	0	A	X	X	‐	X	X	X	Generic information	−0.52 (0.09)	8 (excellent)
Houkes, 2011^23^	CT	126	10	62	27	A	‐	X	‐	X	‐	X	No intervention	−0.41 (0.25)	4 (fair)
Kouwenhoven‐Pasmooij, 2018^29^	cRCT	110	15	57	28	A	X	X	‐	X	X	X	Generic information and personalized letter with feedback	−0.83 (0.48)	7 (good)
Robroek, 2012^28^	cRCT	661	22	33	45	A	‐	X	‐	X	X	‐	Standard lifestyle intervention programme	0.03 (0.06)	6 (good)
Slootmaker, 2009^25^	RCT	87	2	33	65	A	X	‐	‐	X	‐	‐	Generic information	−0.08 (0.18)	8 (excellent)
Strijk, 2012^26^	RCT	606	10	29	61	A‐S	‐	X	X	‐	‐	X	No intervention	0.03 (0.09)	7 (good)
Verweij, 2013^27^	cRCT	460	12	35	53	A	X	X	‐	X	‐	X	Health appraisal and advice	0.05 (0.10)	9 (excellent)
Viester, 2018^20^	RCT	278	67	32	1	A	‐	X	‐	X	X	X	Usual care	−0.24 (0.12)	8 (excellent)
van Wier, 2011^22^	RCT	970	5	35	60	A	Arm 1: X Arm 2: X	Arm 1: X Arm 2: X	‐	Arm 1: X Arm 2: X	Arm 1: X Arm 2: X	Arm 1: ‐ Arm 2: ‐	Generic information	Arm 1: −0.50 (0.10) Arm 2: −0.37 (0.09)	6 (good)
Wierenga, 2014^31^	CT	194	3	17	80	A‐S	‐	‐	X	‐	‐	‐	Usual care	0.20 (0.17)	5 (good)
Total		5183	21	31	48		6	12	4	9	7	9			

^a^ RCT, randomized controlled trial; cRCT, cluster randomized controlled trial; CT, controlled trial.

^b^ *n* concerns the number of participants with BMI information from baseline and follow‐up measurements.

^c^ A, agentic; A‐S, agento‐structural.

^d^ X, applicable; ‐, not applicable.

In seven studies, sufficient participants of all three socio‐economic groups were represented to estimate the effectiveness of the intervention stratified by SEP.^22^, ^23^, ^26^, ^27^, ^28^, ^29^, ^31^ Four studies only had participants in an intermediate and high SEP,^21^, ^24^, ^25^, ^31^ and two studies only had participants from low and intermediate SEP (Table 1).^19^, ^20^ Almost half of the participants had a high SEP (48%), 31% an intermediate SEP and 21% a low SEP. The mean BMI at baseline was 26.75 kg/m^2^ (SD 4.09 kg m^−2^). Most participants were male (63%), and the mean age was 45.72 years (SD 9.46 years).

### 3.1 Overall effects

As shown in Table 2, a small and statistically non‐significant decrease in BMI was found of 0.12 kg/m^2^ (95% CI: −0.01, 0.25) for the intervention groups compared with the control groups. Four out of the 16 interventions were effective compared with the control condition (data not shown).^19^, ^20^, ^22^

**TABLE 2:** Overall intervention effects, intervention * socio‐economic position interaction and effects stratified by socio‐economic position of 16 workplace health promotion interventions in 5183 workers on BMI

	Studies	Participants	Effects on BMI (kg/m^2^)
	*N*	*n*	*β* (95% CI)
Overall intervention effect	16	5392^a^	−0.12 (−0.25, 0.01)
Intervention * socio‐economic position interaction			
Low vs. intermediate socio‐economic position	10		0.06 (−0.15, 0.27)^b^
Low vs. high socio‐economic position	10		0.06 (−0.14, 0.27)
Intermediate vs. high socio‐economic position	14		0.10 (−0.12, 0.32)
Stratified by socio‐economic position			
Low socio‐economic position	10	1080 (21%)	−0.16 (−0.38, 0.07)
Intermediate socio‐economic position	16	1615 (31%)	−0.12 (−0.29, 0.05)
High socio‐economic position	14	2697 (48%)	−0.09 (−0.26, 0.08)

Abbreviation: BMI, body mass index.

^a^ The number of unique participants is *n* = 5183. Because two studies have more than one intervention arm, the control condition is included multiple times in this analysis, increasing the participant number to 5392.

^b^ Interpretation: this interaction term is based on the studies including participants in low socio‐economic position and participants in intermediate socio‐economic position. Compared with participants with an intermediate socio‐economic position, participants in low socio‐economic position had a non‐significant 0.06 lower reduction in BMI after the intervention relative to the control conditions.

### 3.2 Differential effects and effects within socio‐economic groups

No differential intervention effects were found for participants in the low socio‐economic group compared with those in the intermediate or the high socio‐economic group (low vs. intermediate: *β* 0.06, 95% CI: −0.15, 0.27; low vs. high: *β* 0.06, 95% CI: −0.14, 0.27; Table 2). Also, comparing the intermediate socio‐economic group with the high socio‐economic group, no overall interaction effect was found (*β* 0.10; 95% CI: −0.12, 0.32). Three out of 14 interventions had a differential effect in favour of those with a high SEP compared with those with an intermediate SEP,^21^, ^22^ and one study had a differential effect in favour of those with an intermediate SEP compared with a high SEP.^25^

**FIGURE 2:**
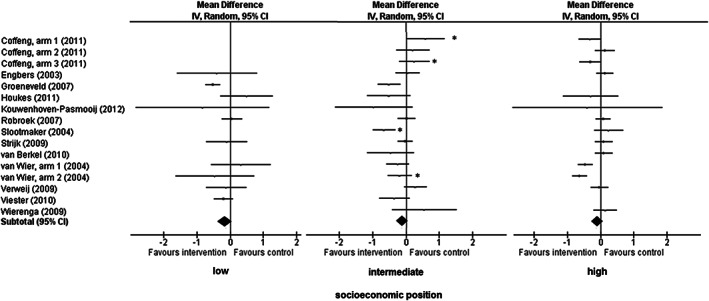
Individual study effects of workplace health promotion programmes on body mass index (BMI), stratified by socio‐economic position (SEP). *intervention * socio‐economic position interaction effects (*p*<0.05) for those with intermediate socio‐economic position compared with high socio‐economic position

Larger reductions in BMI were found for those in low SEP (*β* −0.16, 95% CI: −0.38, 0.07) compared with participants in intermediate (*β* −0.12, 95% CI: −0.29, 0.05) or high SEP (*β* −0.09, 95% CI: −0.26, 0.08; Table 2). As shown in Figure 2, in the low socio‐economic group, only one out of 10 interventions showed a statistically significant reduction in BMI.^19^ In the intermediate^19^, ^25^ and high^22^ socio‐economic group, two out of 16 and 14, respectively, interventions showed a statistically significant reduction in BMI.

### 3.3 Associations of study and intervention characteristics with the effectiveness

Interventions evaluated in an RCT overall showed a statistically significant reduction in BMI (*β* −0.25, 95% CI: −0.43, −0.07), which was not the case for cluster RCTs (*β* 0.03, 95% CI: −0.25, 0.32) and CTs (*β* 0.03, 95% CI: −0.25, 0.32) (Table 3). The association between study design and intervention effectiveness did not differ across socio‐economic groups. Overall, the effectiveness of interventions in which BMI was measured through self‐report did not differ from those interventions in which BMI was measured objectively. Although not statistically significant, studies with a subjective measure of BMI were more effective among workers of low or intermediate SEP than studies with objective BMI measurements. In contrast, among workers with a high SEP, interventions with an objective measure of BMI were more effective than subjectively measured BMI. An interaction between measurement type and intervention was only found between workers in the intermediate and high SEP group but not between the workers with a low SEP and workers with an intermediate or high SEP.

**TABLE 3:** Influence of study and intervention characteristics on the effectiveness of workplace health promotion programmes on body mass index (BMI)

	Total group		Effects of interventions on BMI					
			Low SEP		Intermediate SEP		High SEP	
	*N* studies	*β* (95% CI)	*N* studies	*β* (95% CI)	*N* studies	*β* (95% CI)	*N* studies	*β* (95% CI)
Design								
RCT	7	−0.25 (−0.43, −0.07)	5	−0.29 (−0.54, −0.04)	7	−0.33 (−0.53, −0.14)	5	−0.16 (−0.50, 0.19)
cRCT	6	0.03 (−0.06, 0.12)	3	0.00 (−0.26, 0.26)	6	0.17 (−0.05, 0.39)	6	−0.07 (−0.24, 0.09)
CT	3	0.03 (−0.25, 0.32)	2	0.17 (−0.68, 1.02)	3	−0.04 (−0.51, 0.44)	3	0.11 (−0.09, 0.31)
Between group difference (RCT vs. cRCT/CT)		−0.28 (−0.51, 0.05)		−0.33 (−0.78, 0.13)		−0.44 (−0.74, −0.14)		−0.13 (−0.50, 0.23)
Measurement type								
Objective	8	−0.12 (−0.32, 0.08)	4	0.10 (−0.30, 0.50)	8	0.01 (−0.20, 0.21)	8	−0.21 (−0.45, 0.03)
Subjective	8	−0.12 (−0.30, 0.07)	6	−0.24 (−0.49, 0.01)	8	−0.25 (−0.53, 0.02)	8	0.08 (−0.04, 0.20)
Between group difference		0.00 (−0.28, 0.29)		−0.35 (−0.95, 0.26)		−0.27 (−0.66, 0.12)		0.30 (−0.02, 0.63)
Level of individual agency								
Agento‐structural intervention	5	0.06 (−0.05, 0.17)	1	−0.10 (−0.71, 0.52)	5	0.12 (−0.19, 0.43)	5	0.04 (−0.11, 0.19)
Agentic intervention	11	−0.21 (−0.37, −0.04)	9	−0.16 (−0.40, 0.09)	11	−0.20 (−0.41, −0.00)	9	−0.17 (−0.41, 0.07)
Between group difference		0.27 (0.01, 0.54)		0.06 (−0.88, 1.00)		0.33 (−0.10, 0.76)		0.20 (−0.15, 0.56)
High‐risk approach								
Indicated prevention	6	−0.32 (−0.54, −0.10)	5	−0.38 (−0.65, −0.10)	6	−0.32 (−0.62, −0.01)	5	−0.26 (−0.60, 0.07)
Universal prevention	10	0.01 (−0.07, 0.09)	5	−0.06 (−0.24, 0.13)	10	0.01 (−0.16, 0.18)	9	0.01 (−0.10, 0.13)
Between group difference		−0.32 (−0.55, −0.10)		−0.34 (−0.75, 0.07)		−0.32 (−0.70, 0.06)		−0.29 (−0.62, 0.01)
Type of interventions								
Counselling component	12	−0.19 (−0.34, −0.04)	9	−0.15 (−0.38, 0.09)	12	−0.12 (−0.30, 0.06)	10	−0.20 (−0.40, 0.01)
Noncounselling component	4	0.11 (−0.02, 0.25)	1	−0.40 (−1.59, 0.80)	4	−0.05 (−0.56, 0.46)	4	0.15 (−0.01, 0.31)
Between group difference		−0.29 (−0.59, 0.00)		0.25 (−1.27, 1.77)		−0.04 (−0.53, 0.46)		−0.35 (−0.70, −0.00)
Type of interventions								
Environmental component	6	0.07 (−0.03, 0.17)	2	−0.16 (−0.71, 0.39)	6	0.09 (−0.13, 0.44)	6	0.06 (−0.07, 0.19)
Nonenvironmental component	10	−0.25 (−0.41, −0.08)	8	−0.14 (−0.41, 0.12)	10	−0.23 (−0.45, −0.01)	8	−0.22 (−0.47, 0.03)
Between group difference		0.32 (0.08, 0.55)		−0.03 (−0.85, 0.80)		0.34 (−0.05, 0.73)		0.27 (−0.05, 0.60)
Intervention delivery								
Face‐to‐face	9	−0.15 (−0.33, 0.02)	6	−0.23 (−0.50, 0.04)	9	−0.12 (−0.39, 0.14)	7	−0.08 (−0.23, 0.07)
Other	7	−0.07 (−0.29, 0.14)	4	0.03 (−0.24, 0.29)	7	−0.12 (−0.36, 0.11)	7	−0.07 (−0.35, 0.21)
Between group difference		−0.08 (−0.36, 0.21)		−0.24 (−0.77, 0.29)		−0.01 (−0.43, 0.42)		−0.03 (−0.40, 0.35)
Number of sessions								
>5 sessions	6	−0.30 (−0.54, −0.06)	5	−0.21 (−0.62, 0.21)	6	−0.27 (−0.49, −0.05)	5	−0.24 (−0.60, 0.11)
≤5 sessions	10	0.01 (−0.09, 0.10)	5	−0.12 (−0.35, 0.10)	10	−0.01 (−0.25, 0.23)	9	−0.00 (−0.13, 0.13)
Between group difference		−0.29 (−0.52, −0.05)		−0.14 (−0.70, 0.41)		−0.28 (−0.68, 0.11)		−0.24 (−0.59, 0.12)
Level of provision								
Individual level	9	−0.27 (−0.45, −0.09)	8	−0.14 (−0.41, 0.12)	9	−0.28 (−0.51, −0.06)	7	−0.20 (−0.49, 0.09)
Group level	7	0.06 (−0.03, 0.15)	2	−0.16 (−0.71, 0.39)	7	0.10 (−0.09, 0.29)	7	0.01 (−0.14, 0.15)
Between group difference		−0.32 (−0.55, −0.09)		0.03 (−0.80, 0.85)		−0.40 (−0.75, −0.05)		−0.21 (−0.55, 0.14)

*Note*: Overall effects and stratified by socio‐economic position (SEP) are shown.

For intervention characteristics, agentic interventions, interventions targeted at high‐risk groups, interventions with a counselling component, without an environmental component, more than five sessions and interventions provided at the individual level had a statistically significant reduction in BMI, with betas ranging from −0.19 (95% CI: −0.34, −0.04) (for interventions with a counselling component) to −0.32 kg/m^2^ (95% CI: −0.54, −0.10) (for indicated prevention strategies; Table 3). The influence of these intervention characteristics on the effectiveness did not differ across socio‐economic groups.

## 4 DISCUSSION

No differential effects of workplace health promotion across SEP on BMI were found. In all socio‐economic groups, a small, but statistically non‐significant, decrease in BMI was found. This IPD meta‐analysis showed that interventions evaluated within an RCT, agentic interventions, intervention that focused on a high‐risk group, included a counselling component, consisted of more than five sessions, or offered at the individual level did reduce BMI. However, the reduction in BMI was 0.32 kg/m^2^ or lower.

### 4.1 No differential effectiveness of workplace health promotion programmes on BMI

Theoretically, public health interventions could generate socio‐economic health inequalities in different ways, for example, by differences in delivery, reach and compliance, or by having greater effects among individuals with a high compared with a low SEP.

Concerning the delivery, it is remarkable that most studies focused on intermediate and high educated workers (79% of the IPD sample). The interventions were either more often provided to workers in high SEP or these workers were more likely to participate in offered interventions. Only two studies targeted workers in the construction industry,^19^, ^20^ the majority of these participants had a low SEP. Offering effective interventions mainly to workers in high SEP would lead to intervention‐generated inequalities. In this IPD meta‐analysis, information on reach (initial participation) was lacking or not well defined in the individual studies. However, a systematic review investigating reach of workplace health promotion programmes did not find clear inequalities in reach across socio‐economic groups.^32^

We hypothesized that workplace health promotion programmes would be less effective among workers in low SEP compared with workers in higher SEP. Following the framework for evaluating the impact of obesity prevention strategies on socio‐economic inequalities in body weight, this was in particular expected for agentic interventions as those interventions focus on cognitive‐behavioural strategies to support making independent choices, for example, health education interventions. Our IPD meta‐analysis, however, showed no differential effects on BMI across socio‐economic groups for workplace health promotion programmes. This is in line with several reviews on public health interventions that showed that the majority of the included studies on the prevention of unhealthy behaviour or obesity did not have differential effects across socio‐economic groups.^5^, ^6^, ^7^, ^9^, ^10^, ^14^, ^15^, ^33^ However, as unhealthy behaviours and obesity are more prevalent in workers in low SEP, the need for effective interventions for these workers remains of eminent importance. According to Hillier‐Brown et al.,^34^ implementing effective interventions targeted specifically to individuals in low SEP, for example, blue collar workers, might be effective in reducing the socio‐economic gradient in obesity.

### 4.2 Associations with study design and intervention characteristics

Overall, the reduction in BMI was small and not statistically significant. This is in line with earlier meta‐analyses, which include studies from different countries. Verweij et al. found a pooled effect of −0.34 kg/m^2^ (95% CI: −0.46, −0.22) for RCTs evaluating workplace health promotion interventions.^18^ The seven RCTs in our meta‐analyses had a pooled effect of −0.25 (95% CI: −0.43, −0.07), in contrast to 0.03 (95% CI: −0.06, 0.12) for cluster RCTs and 0.03 (95% CI: −0.25, 0.32) for controlled trials. This is surprising, because other reviews have found larger effects among studies of lower methodological quality or among non‐randomized controlled studies.^17^, ^35^ However, this phenomenon could be explained by the difference in intervention types offered in the different study designs. Two specific interventions were effective, of which one also showed statistically significant, positive effects among workers in low SEP.^19^ These interventions were offered at the individual level, which may be more suitable to be evaluated using an RCT. The current study showed that regardless of SEP, interventions focused on high‐risk groups, with a counselling component, provided at the individual level or with more than five sessions were in general effective. In addition, we noted some insignificant differences between workers in low SEP and high SEP whether subjective or objective measurement of BMI had any effect. It should be noticed that there was overlap between type of measurement of BMI and intervention characteristics. Due to a lack of statistical power, study or intervention characteristics could only be analysed univariate in the meta‐regression model. Therewith, it was not possible to disentangle the study and interventions characteristics that contribute most to a reduction in BMI, and these results should therefore be interpreted with caution.

### 4.3 Need for effective interventions among workers in low SEP

As the majority of the included interventions were not more effective in reducing BMI than control conditions, regardless of the SEP of the participants, this raises the question of which interventions are needed to reduce BMI in low socio‐economic groups or to reduce socio‐economic inequalities in BMI. The studies in this IPD meta‐analysis consisted of agentic or agento‐structural interventions, often counselling in combination with health education. All included interventions required individuals to make independent choices (e.g., free fruit at the workplace, healthier food options at the canteen or food steps to promote stair use). It was hypothesized that in particular, workers in higher socio‐economic groups would benefit from these kinds of interventions. None of the included studies were considered to evaluate structural interventions, while it is expected that such interventions are more likely to be effective among persons in low SEP. The current study showed, however, no evidence for an increase in inequalities after agentic or agento‐structural interventions. Although some interventions contained, to some extent, an environmental change, they could not be considered as structural because individuals still needed to make their own choices. According to the framework for the likely impact of obesity prevention strategies on socio‐economic inequalities in population body weight, structural interventions have more potential to reduce inequalities, because the individual choice is largely removed, such as providing only healthy food options at the canteen.^10^, ^11^ Such interventions would provide a context for healthy behaviour and could be combined with counselling interventions addressing high‐risk groups. However, a first step could be to make a comprehensive analysis of the determinants of the inequalities in health behaviour and BMI and design integrated interventions targeted to workers in low SEP.

### 4.4 Strengths and limitations

After combining original data from 16 interventions of 13 studies into one dataset, and analysing the results across socio‐economic groups, this IPD made it possible to assess socio‐economic inequalities in the effectiveness of workplace health promotion programmes and to provide insight into the association of study design and intervention characteristics with this effectiveness. Most workplace health promotion programmes did measure an indicator of SEP, but analysing differential effects across sociodemographic groups was mostly not performed.

A limitation is that—in contrast to what has been described in the protocol—the influence of reach and work‐related characteristics on the effectiveness of the studied worksite health promotion programmes could not be investigated. This information was not available in most of the included studies or was too heterogeneous to be harmonized, as a result of which these analyses could not be conducted. We recommend to include relevant process information, such as reach and uptake, and information on work‐related characteristics in publications on the effectiveness of workplace health promotion programmes. Work‐related characteristics have been found to be associated with BMI, for example, a higher BMI among workers with an imbalance between perceived high efforts and low rewards at work,^36^, ^37^ and among workers with high physical work demands.^38^, ^39^ The focus of the manuscript is on differential effects of workplace health promotion programmes on BMI across SEP groups. Therefore, only studies with at least multiple SEP groups were included. However, we found one other study that concerned only a single SEP group (high SEP) but met all inclusion criteria.^40^ The effectiveness for this study was comparable with the included interventions. Although in the selection process no studies were identified that were restricted to low SEP workers only, providing tailored and effective interventions to workers in low SEP only could reduce socio‐economic inequalities in BMI. For six studies, no data were available. Five of these six studies were more than 10 years old. Although it is a strength that all studies were performed in the Netherlands within a homogeneous occupational health context, generalization to other contexts should be done with caution. It would be relevant to perform a similar analysis in different countries and compare the results across countries.

## 5 CONCLUSION

In conclusion, small statistically non‐significant intervention effects of workplace health promotion programmes on BMI were found. No evidence was shown for intervention‐generated inequalities in BMI for workplace health promotion programmes. These findings are in line with previous studies showing no differential effectiveness on BMI across socio‐economic groups. Interventions evaluated within an RCT, agentic interventions, interventions focusing on high‐risk groups, with counselling components, more than five sessions or being offered at the individual level did statistically significantly reduce BMI. No evidence was found for intervention‐generated SEP inequalities in BMI.

## CONFLICT OF INTEREST

The institutions of the authors received one grant from Netherlands Organisation for Health Research and Development (ZonMw) during the conduct of the study. Prof. Dr. A. J. van der Beek reports personal fees from spin‐off company Evalua Nederland B.V. and grants from Dutch National Social Security Agency outside the submitted work.

## Supporting information


**Supplementary file A:** Search strategy
Supplementary file B: Overview of excluded studies
