# Supplementary material for: Socio‐economic inequalities in the effectiveness of workplace health promotion programmes on body mass index: An individual participant data meta‐analysis
Source: Obes Rev. 2020 Jul 21;21(11):e13101. doi: 10.1111/obr.13101 (PMC7583467; doi:10.1111/obr.13101)
Supplement: Supplementary file 1 — Supplementary file A: Search strategy Supplementary file B: Overview of excluded studies [file OBR-21-e13101-s001.docx]

**Supplementary file A. Search strategy**

2018 February 19th

**Search strategy in Embase (read from bottom-up).**

| **No** | **Query** |
| --- | --- |
| **#5** | #1 AND #2 AND #3 AND #4 |
| **#4** | 'Netherlands'/exp OR (Netherlands OR dutch):ab,ti,kw,ca,ta,cy,ad |
| **#3** | 'workplace'/exp OR Employee/de OR employer/de OR worker/de OR (worker OR workplace* OR worksite* OR employee* OR employer* OR (work* NEAR/3 population*) OR 'at work'):ab,ti,kw |
| **#2** | 'intervention study'/exp OR 'program evaluation'/exp OR 'education program'/exp OR 'smoking cessation program'/exp OR 'health education'/exp OR (intervention* OR program* OR (health NEAR/3 (education* OR promot*))):ab,ti,kw |
| **#1** | 'lifestyle'/exp OR 'lifestyle modification'/exp OR 'sedentary lifestyle'/exp OR 'physical activity'/exp OR 'sitting'/exp OR exercise/exp OR sport/exp OR 'health behavior'/de OR 'alcohol consumption'/de OR 'drinking behavior'/exp OR 'smoking cessation'/exp OR obesity/exp OR 'body mass'/exp OR 'body weight'/de OR 'body weight change'/exp OR 'body weight control'/exp OR 'body weight gain'/exp OR 'body weight loss'/exp OR 'waist circumference'/de OR 'waist hip ratio'/de OR 'skinfold thickness'/de OR 'body fat'/de OR 'body composition'/de OR 'body distribution'/de OR 'body fat distribution'/de OR 'dietary intake'/exp OR 'healthy diet'/de OR 'unhealthy diet'/de OR 'feeding behavior'/exp OR (lifestyle OR 'life style' OR tobacco OR smoking OR smoker* OR cigarette* OR alcohol* OR addict* OR drinking OR sedentar* OR (physical* NEAR/3 (activ* OR inactiv*)) OR exercis* OR walking OR cycling OR jogging OR sitting OR running OR sport* OR obes* OR overweight* OR (body NEAR/3 (mass OR weight OR fat OR composition* OR distribut*)) OR (weight NEAR/3 (gain OR change OR loss OR reduc*)) OR overweight OR bmi OR (waist NEAR/3 (circumferen* OR hip)) OR (skinfold* NEAR/3 thick*) OR ((diet* OR eating OR feeding OR calor* OR fat) NEAR/3 (intake* OR behav* OR restrict* OR health* OR unhealth*)) OR 'fast food' OR fruit OR vegetable*):ab,ti,kw |

**Search strategy in Medline Ovid (read from bottom-up).**

| **No** | **Query** |
| --- | --- |
| **#5** | #1 AND #2 AND #3 AND #4 |
| **#4** | Netherlands/ OR (Netherlands OR dutch).ab,ti,kw,jn,cp,in |
| **#3** | workplace/ OR (worker OR workplace* OR worksite* OR employee* OR employer* OR (work* ADJ3 population*) OR at work).ab,ti,kw. |
| **#2** | intervention studies/ OR exp program evaluation/ OR education/ OR education.xs. OR exp health education/ OR (intervention* OR program* OR (health ADJ3 (education* OR promot*))).ab,ti,kw. |
| **#1** | exp life style/ OR exp Motor Activity/ OR exp sports/ OR exp health behavior/ OR exp drinking behavior/ OR exp "Tobacco Use"/ OR exp obesity/ OR Body Mass Index/ OR exp Diet/ OR feeding behavior/ OR sports/ OR exp Running/ OR Bicycling/ OR body weight/ OR exp body weight changes/ OR Waist Circumference/ OR Waist-Hip Ratio/ OR skinfold thickness/ OR exp body composition/ OR Energy Intake/ OR healthy diet/ OR (lifestyle OR life style OR tobacco OR smoking OR smoker* OR cigarette* OR alcohol* OR addict* OR drinking OR sedentar* OR (physical* ADJ3 (activ* OR inactiv*)) OR exercis* OR walking OR cycling OR jogging OR sitting OR running OR sport* OR obes* OR overweight* OR (body ADJ3 (mass OR weight OR fat OR composition* OR distribut*)) OR (weight ADJ3 (gain OR change OR loss OR reduc*)) OR overweight OR bmi OR (waist ADJ3 (circumferen* OR hip)) OR (skinfold* ADJ3 thick*) OR ((diet* OR eating OR feeding OR calor* OR fat) ADJ3 (intake* OR behav* OR restrict* OR health* OR unhealth*)) OR fast food OR fruit OR vegetable*).ab,ti,kw. |

**Search strategy in Google scholar (read from bottom-up).**

| **No** | **Query** |
| --- | --- |
| **#1** | lifestyle\|"life style"\|smoking\|alcohol\|"physical activity"\|obesity\|overweight intervention\|program\|promotion worker\|workplace\|worksite\|employee\|employer Netherlands\|dutch |

**Search strategy in Cochrane CENTRAL (read from bottom-up).**

| **No** | **Query** |
| --- | --- |
| **#5** | #1 AND #2 AND #3 AND #4 |
| **#4** | Netherlands OR Dutch |
| **#3** | intervention* OR program* OR (health NEAR/3 (education* OR promot*)):ab,ti |
| **#2** | worker OR workplace* OR worksite* OR employee* OR employer* OR (work* NEAR/3 population*) OR 'at work'):ab,ti |
| **#1** | lifestyle OR 'life style' OR tobacco OR smoking OR smoker* OR cigarette* OR alcohol* OR addict* OR drinking OR sedentar* OR (physical* NEAR/3 (activ* OR inactiv*)) OR exercis* OR walking OR cycling OR jogging OR sitting OR running OR sport* OR obes* OR overweight* OR (body NEAR/3 (mass OR weight OR fat OR composition* OR distribut*)) OR (weight NEAR/3 (gain OR change OR loss OR reduc*)) OR overweight OR bmi OR (waist NEAR/3 (circumferen* OR hip)) OR (skinfold* NEAR/3 thick*) OR ((diet* OR eating OR feeding OR calor* OR fat) NEAR/3 (intake* OR behav* OR restrict* OR health* OR unhealth*)) OR 'fast food' OR fruit OR vegetable*):ab,ti |

**Search strategy in Web of science (read from bottom-up).**

| **No** | **Query** |
| --- | --- |
| **#5** | #1 AND #2 AND #3 AND #4 |
| **#4** | Netherlands OR dutch |
| **#3** | intervention* OR program* OR (health NEAR/2 (education* OR promot*)) |
| **#2** | worker OR workplace* OR worksite* OR employee* OR employer* OR (work* NEAR/2 population*) OR "at work" |
| **#1** | lifestyle OR "life style" OR tobacco OR smoking OR smoker* OR cigarette* OR alcohol* OR addict* OR drinking OR sedentar* OR (physical* NEAR/2 (activ* OR inactiv*)) OR exercis* OR walking OR cycling OR jogging OR sitting OR running OR sport* OR obes* OR overweight* OR (body NEAR/2 (mass OR weight OR fat OR composition* OR distribut*)) OR (weight NEAR/2 (gain OR change OR loss OR reduc*)) OR overweight OR bmi OR (waist NEAR/2 (circumferen* OR hip)) OR (skinfold* NEAR/2 thick*) OR ((diet* OR eating OR feeding OR calor* OR fat) NEAR/2 (intake* OR behav* OR restrict* OR health* OR unhealth*)) OR "fast food" OR fruit OR vegetable* |

**Search strategy in Web of science (read from bottom-up).**

| **No** | **Query** |
| --- | --- |
| **#5** | #1 AND #2 AND #3 AND #4 |
| **#4** | Netherlands OR dutch |
| **#3** | intervention* OR program* OR (health NEAR/2 (education* OR promot*)) |
| **#2** | worker OR workplace* OR worksite* OR employee* OR employer* OR (work* NEAR/2 population*) OR "at work" |
| **#1** | lifestyle OR "life style" OR tobacco OR smoking OR smoker* OR cigarette* OR alcohol* OR addict* OR drinking OR sedentar* OR (physical* NEAR/2 (activ* OR inactiv*)) OR exercis* OR walking OR cycling OR jogging OR sitting OR running OR sport* OR obes* OR overweight* OR (body NEAR/2 (mass OR weight OR fat OR composition* OR distribut*)) OR (weight NEAR/2 (gain OR change OR loss OR reduc*)) OR overweight OR bmi OR (waist NEAR/2 (circumferen* OR hip)) OR (skinfold* NEAR/2 thick*) OR ((diet* OR eating OR feeding OR calor* OR fat) NEAR/2 (intake* OR behav* OR restrict* OR health* OR unhealth*)) OR "fast food" OR fruit OR vegetable* |

**Supplementary file B. Overview of excluded studies (n=21)**

| **Study**  **#** | **References** | **Reason for exclusion** |
| --- | --- | --- |
| 1 | Proper K, van der Beek AJ, Hildebrandt V, Twisk J, van Mechelen W. Worksite health promotion using individual counselling and the effectiveness on sick leave; results of a randomised controlled trial. Occup Environ Med 2004; 61: 275–279.  - Proper KI, Hildebrandt VH, van der Beek AJ, Twisk JW, van Mechelen W. Effect of individual counseling on physical activity fitness and health: a randomized controlled trial in a workplace setting. Am J Prev Med 2003; 24: 218-26.  Proper KI, de Bruyne MC, Hildebrandt VH, van der Beek AJ, Meerding WJ, van Mechelen W. Costs, benefits and effectiveness of worksite physical activity counseling from the employer's perspective. Scand J Work Environ Health 2004; 30: 36-46. | No data available |
| 2 | - Kwak L, Kremers SP, Werkman A, Visscher TL, van Baak MA, Brug J. The NHF-NRG In Balance-project: the application of Intervention Mapping in the development, implementation and evaluation of weight gain prevention at the worksite. Obes Rev 2007; 8:347-361. - Kwak L, Kremers SP, Candel MJ, Visscher TL, Brug J, van Baak MA. Changes in skinfold thickness and waist circumference after 12 and 24 months resulting from the NHF-NRG In Balance-project. Int J Behav Nutr Phys Act 2010; 7: 26. - Kwak L, Kremers SP, Visscher TL, van Baak MA, Brug J. Behavioral and cognitive effects of a worksite-based weight gain prevention program: the NHF-NRG in balance-project. J Occup Environ Med 2009; 51: 1437-1446. | No data available |
| 3 | - De Kraker H, Hendriksen I, Hildebrandt V, de Korte E, van Maas ED. The effect of a campaign to stimulate walking during lunch break on the physical activity behavior of employees. Geneeskd Sport 2005, 38: 172-178. | No data available |
| 4 | - Maes S, Kittel F, Scholten H, Verhoeven C. Healthier Work at Brabantia', a comprehensive approach to wellness at the worksite. Saf Sci 1992; 15: 351-366. - Maes S, Verhoeven C, Kittel F, Scholten H. Effects of a Dutch work-site wellness-health program: The Brabantia project. Am J Public Health 1998, 88: 1037-1041. | No data available |
| 5 | - Colkesen EB, Ferket BS, Tijssen JGP, Kraaijenhagen RA, van Kalken CK, Peters RJG. Effects on cardiovascular disease risk of a web-based health risk assessment with tailored health advice: A follow-up study. Vasc Health Risk Manage 2011; 7: 67-74. | No data available |
| 6 | - Willemsen MC, de Vries H, van Breukelen G, Genders R. Long-term effectiveness of two Dutch work site smoking cessation programs. Health Educ Behav 1998; 25: 418-435. - Willemsen MC, de Vries H. Evaluation of a smoking cessation intervention for Dutch employees consisting of self help methods and a group programme. Tobacco control; 1995; 4: 351-364. | No data available |
| 7 | - Vermeer WM, Leeuwis FH, Koprulu S, Zouitni O, Seidell JC, Steenhuis IH. The process evaluation of two interventions aimed at portion size in worksite cafeterias. J Hum Nutr Diet 2012; 25: 180-188. - Vermeer WM, Steenhuis IH, Leeuwis FH, Heymans MW, Seidell JC. Small portion sizes in worksite cafeterias: do they help consumers to reduce their food intake? Int J Obes 2011; 35: 1200-1207. - Vermeer WM, Alting E, Steenhuis IH, Seidell JC. Value for money or making the healthy choice: the impact of proportional pricing on consumers' portion size choices. Eur J Public Health 2010; 20: 65-69. | No suitable data (no individual participant data) |
| 8 | - Brug J, Steenhuis I, van Assema P, de Vries H. The impact of a computer-tailored nutrition intervention. Preventive Medicine 1996; 25: 236-242. - Brug J. Dutch research into the development and impact of computer-tailored nutrition education. Eur J Clin Nutr 1999; 53: S78-S82. | No suitable data (no individual participant data) |
| 9 | - Laan EK, Kraaijenhagen RA, Peek N, Busschers WB, Deutekom M, Bossuyt PM, Stronks K, Essink-Bot ML. Effectiveness of a web-based health risk assessment with individually-tailored feedback on lifestyle behaviour: study protocol. BMC Public Health 2012; 12: 200. | No suitable data (raw data that could not be linked) |
| 10 | - Brandt van den FA, Nagelhout GE, Winkens B, Evers SM, Kotz D, Chavannes NH, van Schayck CP. The effect of financial incentives on top of behavioral support on quit rates in tobacco smoking employees: study protocol of a cluster-randomized trial. BMC Public Health 2016; 16: 1056. | Data not yet available (study in progress) |
| 11 | - Velema E, Vyth EL, Hoekstra T, Steenhuis IHM. Nudging and social marketing techniques encourage employees to make healthier food choices: a randomized controlled trial in 30 worksite cafeterias in The Netherlands. Am J Clin Nutr 2018; 107:236-246. - Velema E, Vyth EL, Steenhuis IH. Using nudging and social marketing techniques to create healthy worksite cafeterias in the Netherlands: intervention development and study design. BMC Public Health 2017; 17: 63. | Data not yet available (study in progress). |
| 12 | - Valk de, RH. [Dietary intervention in occupational medicine] Dutch,Ned Tijdschr Geneeskd, 134: 338-341. | Researchers could not be reached |
| 13 | - Hendriksen IJM, Zuiderveld B, Kemper HCG, Bezemer PD. Effect of commuter cycling on physical performance of male and female employees. Med Sci Sports Exerc 2000, 32: 504-510. | No data on SEP |
| 14 | - Vyth EL, Steenhuis IHM, Heymans MW, Roodenburg AJC, Brug J, Seidell JC. Influence of Placement of a Nutrition Logo on Cafeteria Menu Items on Lunchtime Food Choices at Dutch Work Sites. J Am Diet Assoc 2011, 111: 131-136. | No relevant outcomes |
| 15 | - Gärtner FR, Nieuwenhuijsen K, Ketelaar SM, van Dijk FJ, Sluiter JK. The mental vitality @ work study: effectiveness of a mental module for workers' health surveillance for nurses and allied health care professionals on their help-seeking behavior. [J Occup Environ Med.](https://www.ncbi.nlm.nih.gov/pubmed/24064780) 2013; 55(10):1219-1229. | No relevant outcomes |
| 16 | - Niessen MAJ, Kraaijenhagen RA, Dijkgraaf MGW, Van Pelt D, Van Kalken CK, Peek N. Impact of a web-based worksite health promotion program on absenteeism. J Occup Environ Med 2012; 54: 404-408 | No relevant outcomes |
| 17 | - De Vries JD, van Hooff ML, Geurts SA, Kompier MA. Efficacy of an exercise intervention for employees with work-related fatigue: study protocol of a two-arm randomized controlled trial. BMC Public Health 2015; 15:1117. - De Vries JD, van Hooff ML, Geurts SA, Kompier MA. Exercise to reduce work-related fatigue among employees: A randomized controlled trial. Scand J Work Environ Health 2017; 43: 337-349. | No relevant outcomes |
| 18 | - Brug J, Steenhuis I, van Assema P, Glanz K, De Vries H. Computer-tailored nutrition education: differences between two interventions. Health Educ Res 1999; 14: 249-56. | No follow-up information on BMI |
| 19 | - Oenema A, Tan F, Brug J. Short-Term Efficacy of a Web-Based Computer-Tailored Nutrition Intervention: Main Effects and Mediators. Ann Behav Med 2005;29:54-63. | No follow-up information on BMI |
| 20 | - Steenhuis I, van Assema P, van Breukelen G, Glanz K, Kok G, de Vries H. The impact of educational and environmental interventions in Dutch worksite cafeterias. Health Promot Int 2004; 19: 335-43. | No follow-up information on BMI |
| 21 | - van Drongelen A, van der Beek AJ, Hlobil H, Smid T, Boot CR. Development and evaluation of an intervention aiming to reduce fatigue in airline pilots: design of a randomised controlled trial. BMC Public Health 2013; 13: 776. - van Drongelen A, Boot CR, Hlobil H, Twisk JW, Smid T, van der Beek AJ. Evaluation of an mHealth intervention aiming to improve health-related behavior and sleep and reduce fatigue among airline pilots. Scand J Work Environ Health 2014;40:557-68. - van Drongelen A, Boot CR, Hlobil H, Smid T, van der Beek AJ. Process evaluation of a tailored mobile health intervention aiming to reduce fatigue in airline pilots. BMC Public Health 2016;16:894. | Only participants with a high SEP |
